# Supplementary material for: The Autocrine Role of Placental Extracellular Vesicles from Missed Miscarriage in Causing Senescence: Possible Pathogenesis of Missed Miscarriage
Source: Cells. 2022 Dec 1;11(23):3873. doi: 10.3390/cells11233873 (PMC9740889; doi:10.3390/cells11233873)
Supplement: Supplementary file 1 [file cells-11-03873-s001.zip › cells-1926732 supple Table 1-2.pdf]

Table S1 Abundantly expressed miRNAs in placental large EVs

**Up regulated miRNA(n=18)**	**Down regulated miRNA(n=36)**
hsa-miR-125b-1-3p	hsa-miR-133a-3p
hsa-miR-584-5p	hsa-miR-512-5p
hsa-miR-1290	hsa-miR-519e-5p
hsa-miR-3614-5p	hsa-miR-376c-3p
hsa-miR-4661-5p	hsa-miR-496
hsa-miR-3690	hsa-miR-377-5p
hsa-miR-549a-3p	hsa-miR-369-3p
hsa-miR-24-2-5p	hsa-miR-1185-5p
hsa-miR-330-5p	hsa-miR-376c-5p
hsa-miR-223-5p	hsa-miR-380-3p
hsa-miR-3918	hsa-miR-337-3p
hsa-miR-1246	hsa-miR-373-3p
hsa-miR-1301-3p	hsa-miR-145-5p
hsa-miR-100-5p	hsa-miR-376b-5p
hsa-miR-24-1-5p	hsa-miR-876-3p
hsa-miR-181a-2-3p	hsa-miR-143-5p
hsa-miR-511-5p	hsa-miR-483-3p
hsa-miR-192-5p	hsa-miR-12136
	hsa-miR-126-5p
	hsa-miR-1197
	hsa-miR-154-5p
	hsa-miR-376a-3p
	hsa-miR-675-3p
	hsa-miR-539-3p
	hsa-miR-1-3p
	hsa-miR-133b
	hsa-miR-655-3p
	hsa-miR-873-3p
	hsa-miR-487b-3p
	hsa-miR-199a-5p
	hsa-miR-214-3p
	hsa-miR-378a-5p
	hsa-miR-199b-3p
	hsa-miR-199a-3p
	hsa-miR-519b-3p
	hsa-miR-665

Table S2 Abundantly expressed miRNAs in placental small EVs

Up-regulated miRNAs (n=35)	Down-regulated miRNAs (n=59)
hsa-miR-1246	hsa-miR-133a-3p
hsa-miR-1290	hsa-miR-376c-3p
hsa-miR-584-5p	hsa-miR-376a-3p
hsa-miR-125b-1-3p	hsa-miR-380-3p
hsa-miR-181a-2-3p	hsa-miR-496
hsa-miR-330-5p	hsa-miR-486-3p
hsa-miR-6510-3p	hsa-miR-655-3p
hsa-miR-24-3p	hsa-miR-372-5p
hsa-miR-3934-5p	hsa-miR-541-3p
hsa-miR-6715b-3p	hsa-miR-512-5p
hsa-miR-3065-5p	hsa-miR-199a-5p
hsa-miR-24-2-5p	hsa-miR-3613-5p
hsa-miR-1910-5p	hsa-miR-19b-3p
hsa-miR-3614-5p	hsa-miR-337-3p
hsa-miR-22-5p	hsa-miR-133b
hsa-miR-3925-5p	hsa-miR-495-3p
hsa-miR-125b-5p	hsa-miR-206
hsa-miR-4800-3p	hsa-miR-1185-5p
hsa-miR-4728-3p	hsa-miR-126-5p
hsa-miR-549a-3p	hsa-miR-372-3p
hsa-miR-9-5p	hsa-miR-302a-5p
hsa-miR-338-5p	hsa-miR-519e-5p
hsa-miR-1299	hsa-miR-218-5p
hsa-miR-508-3p	hsa-miR-381-3p
hsa-miR-937-3p	hsa-miR-519d-3p
hsa-miR-100-5p	hsa-miR-518a-3p
hsa-miR-191-3p	hsa-miR-1296-5p
hsa-miR-3189-3p	hsa-miR-126-3p
hsa-miR-6747-3p	hsa-miR-520g-3p
hsa-miR-1301-3p	hsa-miR-1197
hsa-miR-107	hsa-miR-451a
hsa-miR-27a-3p	hsa-miR-335-3p
hsa-miR-3605-3p	hsa-miR-487b-3p
hsa-miR-2116-3p	hsa-miR-490-5p
hsa-miR-450a-1-3p	hsa-miR-520h
	hsa-miR-127-5p
	hsa-miR-202-5p
	hsa-miR-1323
	hsa-miR-1289
	hsa-miR-876-3p
	hsa-miR-526a-3p
	hsa-miR-193a-3p
	hsa-miR-143-5p

	hsa-miR-526b-3p
	hsa-miR-490-3p
	hsa-miR-654-3p
	hsa-miR-296-3p
	hsa-miR-154-5p
	hsa-miR-1271-5p
	hsa-miR-515-5p
	hsa-miR-30b-5p
	hsa-miR-383-5p
	hsa-miR-539-5p
	hsa-miR-665
	hsa-miR-518b
	hsa-miR-377-5p
	hsa-miR-376a-2-5p
	hsa-miR-3179
	hsa-miR-148a-3p
