# Supplementary material for: The Autocrine Role of Placental Extracellular Vesicles from Missed Miscarriage in Causing Senescence: Possible Pathogenesis of Missed Miscarriage
Source: Cells. 2022 Dec 1;11(23):3873. doi: 10.3390/cells11233873 (PMC9740889; doi:10.3390/cells11233873)

Figure legends

Figure S1: KEGG analysis for large EVs showing top 20 pathways

Figure S2: KEGG analysis for small EVs showing top 20 pathways

Figure S3: GO analysis of biological process for large EVs showing top 20 functions

Figure S4: GO analysis of biological process for small EVs showing top 20 functions

Figure S1: KEGG analysis for large EVs

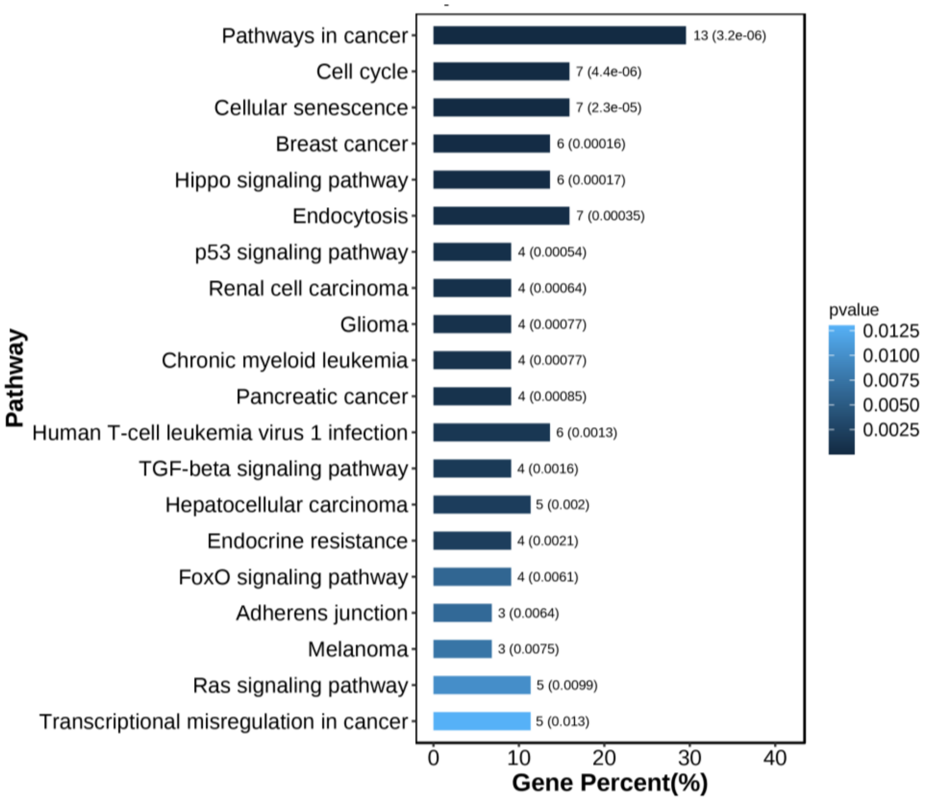

Figure S2: KEGG analysis for small EVs

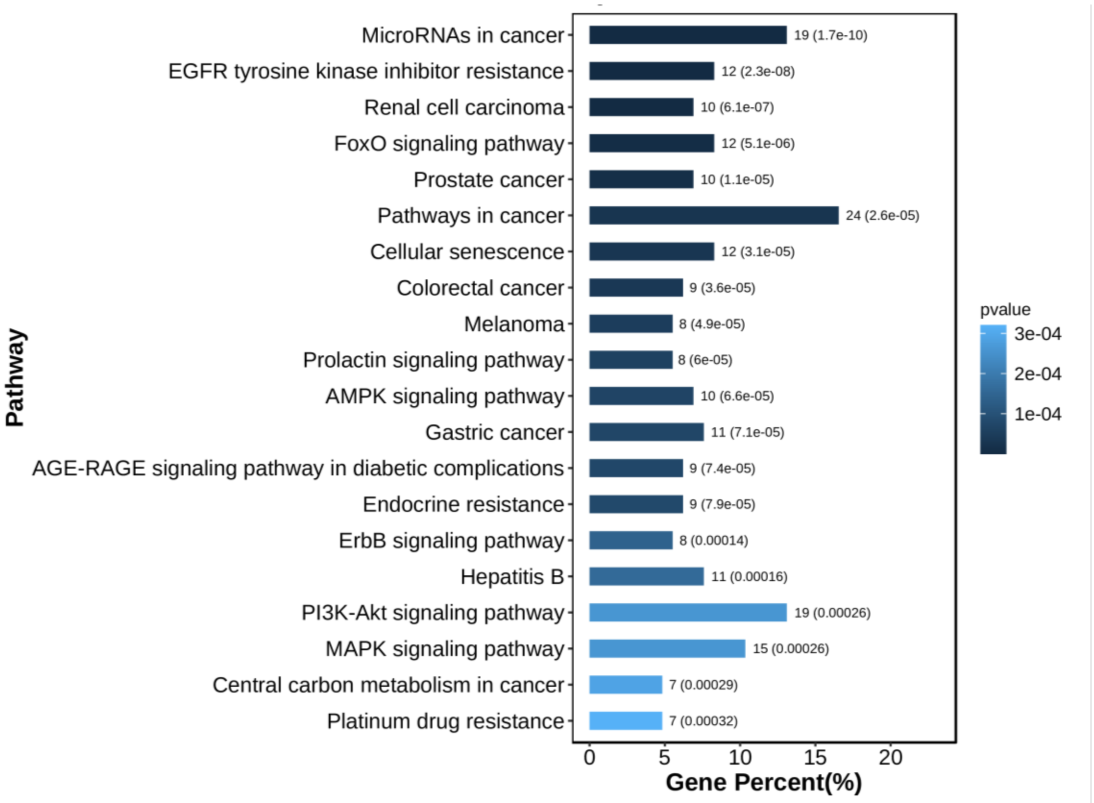

Figure S3: GO analysis of biological process for large EVs

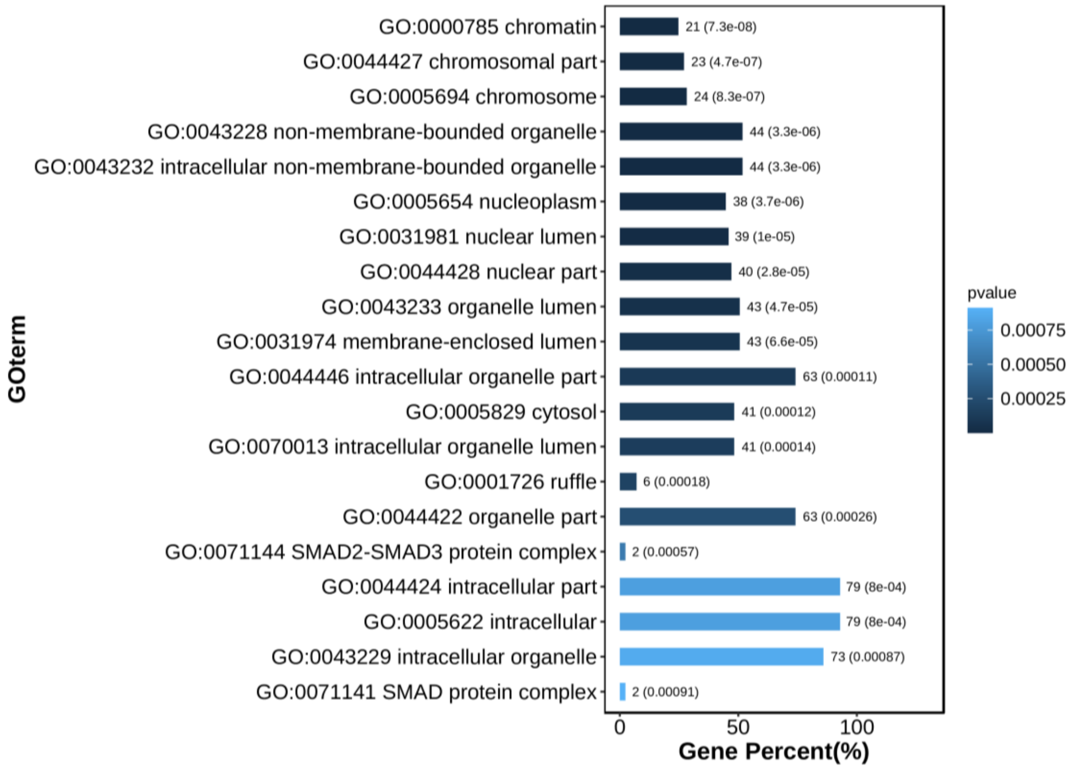

Figure S4: GO analysis of biological process for small EVs

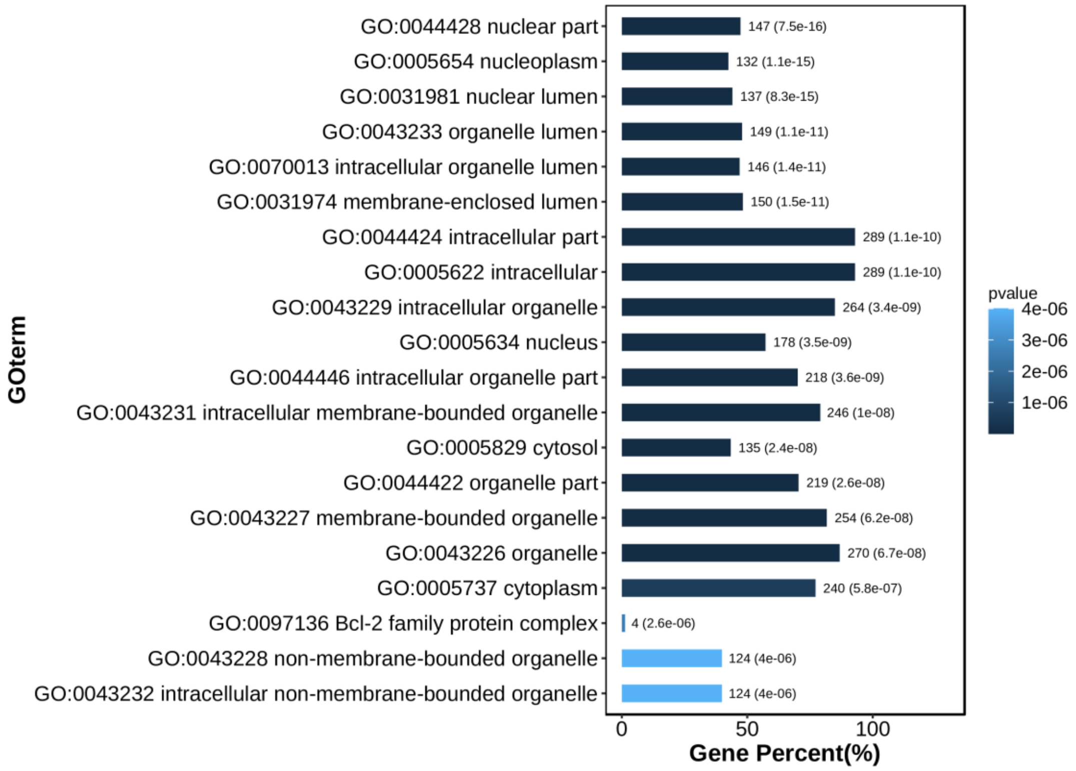

Supplement: Supplementary file 1 [file cells-11-03873-s001.zip › cells-1926732-supplementary-figures.pdf]
